# The impact of Hunter syndrome (mucopolysaccharidosis type II) on health-related quality of life

**DOI:** 10.1186/1750-1172-8-101

**Published:** 2013-07-10

**Authors:** Mireia Raluy-Callado, Wen-Hung Chen, David A H Whiteman, Juanzhi Fang, Ingela Wiklund

**Affiliations:** 1 Evidera, 26-28 Hammersmith Grove, London W6 7HA, UK; 2 Evidera, 7101 Wisconsin Avenue, Suite 600, Bethesda, MD 20814, USA; 3 Shire HGT, 300 Shire Way, Lexington, MA 02421, USA

**Keywords:** Hunter syndrome, Mucopolysaccharidosis type II, Lysosomal storage disease, Patient-reported outcomes, Health-related quality of life

## Abstract

**Background:**

Hunter syndrome (mucopolysaccharidosis type II (MPS II)) is a rare metabolic disease that can severely compromise health, well-being and life expectancy. Little evidence has been published on the impact of MPS II on health-related quality of life (HRQL). The objective of this study was to describe this impact using the Hunter Syndrome-Functional Outcomes for Clinical Understanding Scale (HS-FOCUS) questionnaire and a range of standard validated questionnaires previously used in paediatric populations.

**Methods:**

Clinical and demographic characteristics collected in a clinical trial and responses to four HRQL questionnaires completed both by patients and parents prior to enzyme replacement treatment were used. The association between questionnaire scores and clinical function parameters were tested using Spearman rank-order correlations. Results were compared to scores in other paediatric populations with chronic conditions obtained through a targeted literature search of published studies.

**Results:**

Overall, 96 male patients with MPS II and their parents were enrolled in the trial. All parents completed the questionnaires and 53 patients above 12 years old also completed the self-reported versions. Parents’ and patients’ responses were analysed separately and results were very similar. Dysfunction according to the HS-FOCUS and the CHAQ was most pronounced in the physical function domains. Very low scores were reported in the Self Esteem and Family Cohesion domains in the CHQ and HUI3 disutility values indicated a moderate impact. Scores reported by patients and their parents were consistently lower than scores in the other paediatric populations identified (except the parent-reported Behaviour score); and considerably lower than normative values.

**Conclusions:**

This study describes the impact on HRQL in patients with MPS II and provides a broader context by comparing it with that of other chronic paediatric diseases. Physical function and the ability to perform day-to-day activities were the most affected areas and a considerable impact on the psychological aspects of patients’ HRQL was also found, with a higher level of impairment across most dimensions (particularly Pain and Self Esteem) than that of other paediatric populations. Such humanistic data provide increasingly important support for establishing priorities for health care spending, and as a component of health economic analysis.

## Background

Hunter syndrome is a rare metabolic disease that can severely compromise health, well-being and life expectancy. Also called mucopolysaccharidosis type II (MPS II), it has an X-linked recessive inheritance pattern, occurs primarily in males [1] (with an estimated incidence of 1 per 162,000 births) and is classified as an orphan disease [2]. It is caused by a deficiency of iduronate-2-sulfatase (I2S), a lysosomal enzyme that cleaves O-linked sulfate moieties from the glycosaminoglycans (GAGs) dermatan sulphate and heparan sulphate, the first step in their degradative pathway. This abnormality leads to accumulation of GAGs within almost all tissues and organs, resulting in the multisystem manifestations of MPS II.

The clinical picture includes distinctive facial features, relatively large weight and length parameters at birth, and a protuberance of the tongue that can impair both swallowing and the ability to speak clearly [3,4]. Hearing impairment is also common, with one study finding that 24% of patients had mild hearing loss, 31% moderate and 22% severe (using the World Health Organization International Classification of Impairment, Disability and Handicap [WHO-ICIDH] classification) [5,6]; this problem may go undetected, with potentially major adverse consequences for speech, language and cognitive development. The continuing build-up of GAGs in organs leads to a decline in cardiac function, breathing problems during sleep, abdominal distension, and joint stiffness that affects walking and manual dexterity. Early childhood may see the emergence of an inability to keep up with peers in activities requiring physical exertion. Later, the ability to walk even short distances may be lost and many patients eventually become wheelchair-dependent. The progressive decrease in joint mobility may prevent affected individuals from independently performing self-care (e.g., dressing, toilet care and personal grooming). Also, MPS II is associated with chronic, severely diminished endurance that hinders performance of day-to-day activities.

Although MPS II is characterised by such clinical features, its presentation, severity and prognosis are heterogeneous. For example, in the so-called ‘severe’ phenotype, onset (typically at 2 to 4 years of age) with somatic features is followed by neurological problems and cognitive impairment by the age of 6 years, and death within the first two decades of life (usually from obstructive airway disease or cardiac failure). By contrast, while the ‘attenuated’ phenotype may be associated with somatic features, patients with it generally have little neurological involvement, are of normal intelligence and usually survive into adulthood [7].

Traditionally, treatment for MPS II has targeted the symptoms, with the aim of providing palliative benefit. However, in recent years, clinical trials have demon-strated improvements in selected laboratory markers, certain somatic features and measures of clinical function with the use of enzyme replacement therapy (ERT) comprising regular intravenous administration of idursulfase, a recombinant form of human I2S. While these results are important, it is unclear to what extent they are mirrored by improvements in health-related quality of life (HRQL) [7-10]. In addition, to date, little evidence has been published on the nature and severity of the effect of MPS II on humanistic measures such as HRQL [11,12] – a significant knowledge gap given the disabling and life-limiting effects of the disease.

The objective of this study was, therefore, to describe the effect of MPS II on HRQL to increase understanding of the disease and the need for treatment to be initiated in the clinical setting. From the patient and family perspective, highlighting the impact of MPS II on HRQL may help to identify specific areas with difficulties they might encounter in order to better maintain functioning and to provide the most appropriate support.

## Methods

Data collected between September 2003 and March 2004 at the time of enrolment into a clinical trial [13] were used to describe the impact of MPS II on HRQL. Participants were male patients with biochemical and clinical evidence of MPS II and a forced vital capacity (FVC) of < 80% of predicted and their parents/caregivers. Data used in this study included patient characteristics, clinical markers and HRQL measures at the time of trial enrolment. Parents/caregivers of patients of all ages responded to the parent-reported versions of the HRQL measures, and only patients aged 12 years or older responded to the self-reported versions. At the time of this study, patients received treatment for joint pain and other indications but none of them ERT.

Results were then compared with published data using the same questionnaires in other paediatric populations with chronic conditions such as diabetes (type I), attention deficit hyperactivity disorder, asthma and juvenile idiopathic arthritis as well as published normative values. Similar to the current study, the questionnaires had been completed by patients themselves or by their caregivers (e.g., parents). This comparison was conducted in order to provide a broader context of the burden of illness of suffering from MPS II.

### HRQL Measures

The HRQL measures of this study included one disease-specific and three generic questionnaires, and both the self-reported and parent/caregiver versions:

#### Hunter Syndrome-Functional Outcomes for Clinical Understanding Scale (HS-FOCUS)

The Hunter Syndrome-Functional Outcomes for Clinical Understanding Scale (HS-FOCUS) was developed to capture the impact of MPS II on function [unpublished observations]. The final patient and parent versions of the instrument contain six domains measuring functional status (Walking/Standing; Reach/Grip; Sleeping; Schooling/Work; Activities; Breathing) and two additional domains measuring Satisfaction and Botheredness with the identified function domains. These were not used in the analyses as assessed by clinical experts because they did not accurately reflect the level of satisfaction or botheredness [14]. Each item is scored from 0 to 4, with 0 signifying an ability to complete the activity-related functions ‘without ANY difficulty’ and 4 as being ‘UNABLE to do so’. Complete item responses are averaged to calculate the six individual function domain scores and the Overall Function score, with higher scores representing a higher degree of incapacity. If more than half of the items for a function domain are missing or marked ‘not applicable’, that domain score is deemed to be ‘missing’ and not included in the analysis.

#### The Childhood Health Assessment Questionnaire (CHAQ)

The Childhood Health Assessment Questionnaire (CHAQ) was initially developed for assessing juvenile idiopathic arthritis, from the perspective of the parent or patient, and has been previously applied to other chronic disabling conditions such as MPS II [15]. It is a 30-item instrument that measures functional capacity and independence in activities of daily life across eight domains: Dressing and Grooming; Getting Up; Eating; Walking; Reach; Grip; Hygiene and Activities. For each domain, there is a 4-level difficulty scale that is scored from 0 to 3, with 0 corresponding to ‘without any difficulty’ and 3 to ‘unable to do’. An additional question asks for any aids or devices children usually use for any of the above activities, which is used to adjust the scores of the domains into the CHAQ disability index score (CHAQ DIS). This is used as the outcome measure of functional disabilities in severely disabled patients, with a scale from 0 to 3, where 3 represents the worst functions. CHAQ also presents two visual analogue scales for pain evaluation and overall well-being evaluation, where 0 represents ‘no pain’ and ‘doing very well’ and 100 represents ‘very severe pain’ and feeling ‘very poor’.

#### Childhood Health Questionnaire (CHQ)

The Child Health Questionnaire (CHQ) is a family of generic quality-of-life instruments designed to assess physical and psychosocial health status for children and is normalised for those aged 5–18 years [16]. The reliability and validity of the CHQ has been extensively documented across a wide range of conditions, including MPS II [17] and other rare disorders such as the evaluation of ERT in Niemann-Pick disease. The version for children (CHQ-CF87) includes 12 health concepts: 10 multi-item scales [Physical Functioning (PF); Role/Social–Emotional (RE); Role/Social–Behavioural (RB); Role/Social–Physical (RP); Bodily Pain (BP); General Behaviour (BE); Mental Health (MH); Self Esteem (SE); General Health Perceptions (GH); Family Activities (FA)] and two single-item scales: Change in Health (CH) and Family Cohesion (FC). The parent or caregiver form (CHQ-PF50) includes 14 health concepts and HRQL impacts, comprising those in the children version plus two additional multi-item scales: Parental Impact–Emotional (PE) and Parental Impact–Time (PT). Individual scale scores can be analysed separately, or combined to derive an overall physical and psychosocial score, termed PhS and PsS, respectively. Scores range from 0 to 100, with higher scores reflecting better health.

#### Health Utilities Index (HUI)

The Health Utilities Index (HUI) includes both self and proxy-assessed forms and is part of a family of generic health profiles and preference-based systems intended for describing the following: 1) the experience of patients undergoing therapy; 2) long-term outcomes associated with disease or therapy; 3) the efficacy, effectiveness and efficiency of health care interventions; and 4) the health status of general populations. HUI currently consists of two systems, HUI2 and HUI3. This study used HUI3, the more detailed descriptive system of the two, with full structural independence, and normative population available [18]. The scoring systems provide utility (preference) scores on a scale on which death = 0.00 and perfect health = 1.00. The health status classification and HRQL scoring systems are generic in terms of applying to all people aged 5 years and older in both clinical and general populations. HUI3 has eight attributes (Vision; Hearing; Speech; Ambulation; Dexterity; Emotion; Cognition; Pain), with either five or six levels per attribute. Data can be summarised by attribute using the single-attribute HUI3 utility scores plus by the overall HRQL utility score.

### Clinical measures

The following objective clinical measures collected in the clinical trial were also used to assess the validity of the above-mentioned HRQL measures in describing the effect of MPS II on patients’ HRQL.

#### Forced Vital Capacity (FVC)

Pulmonary function in terms of FVC was determined by spirometry at the time of first ERT infusion. The highest FVC reading of two measurements, reflecting the patient’s better effort of the two tests, was used for analyses. The percentage of predicted FVC was used instead of the absolute values (measured in litres), since the latter are affected by extrapulmonary factors such as age, height and gender, and cannot be compared between patients. Results nearest to 100% of the predicted value represent the most normal function, and in the trial, patients’ impairment of FVC was classified as mild (between 70% and < 80%), moderate to moderate severe (between 50% and 70%), and severe to very severe (less than 50%).

#### Six-minute Walk Test (6MWT)

Patients underwent two measurements of the six-minute walk test (6MWT) at the time of first ERT infusion. The furthest distance walked in the two tests (reported in metres) was used in all analyses, to reflect the patient’s best physical performance. According to the 6MWT results, a severity score of 1 was assigned if the distance was ≥ 500 m (mild to normal severity), 2 if it was ≥ 300 m to < 500 m (moderate severity), and 3 if it was < 300 m (severe).

#### Joint Range of Motion (JROM)

Range of motion tests were employed to quantify the musculoskeletal impairment associated with MPS II. The joint range of motion (JROM) refers to the distance and direction a joint can move to its full potential. Each specific joint has a normal range of motion measurable in degrees by using a goniometer. At baseline, a total of 23 motions were measured for the shoulder, elbow, wrist, index finger, hip and ankle summarised in a global JROM score. The measurements were performed two times at separate visits. For each side of the body the arithmetic mean for the two measurements at each visit was calculated and the mean of these means was used in the analysis. For the current analyses, the global JROM scoring system was used (since individual joints can be variably affected among patients), with range of motion being reported as a percentage, with 100% indicating no limitation in the joint motion.

### Statistical analysis

Responses to the patient self-reported and parent-reported study measures were analysed separately. Demographic and clinical characteristics of patients were first investigated. Mean and standard deviation of each domain and overall scores of both the patient self-reported and parent-reported versions of all study measures were examined separately, with the percentage of missing values. Spearman rank-order correlations were used to test the association between domain scores of all study measures and the patient clinical function values. Data for comparison from other similar paediatric populations were obtained from published studies. A targeted literature search was conducted to identify studies that had used the same HRQL measures in healthy children and adolescents or in those who had other chronic conditions. Data were analysed using Stata version 10.0 statistical software (Stata, College Station, TX, USA).

## Results

Overall, 96 male patients and their parents were included in this study. Participants in the trial were recruited from sites in the United States (35%), United Kingdom (23%), Brazil (22%) and Germany (20%).

Average age of the 96 patients was 14 years old, most of them (82%) reported white ethnicity and were short for their age. The MPS II diagnosis was confirmed on average 10 years ago. Mean disease severity score (defined as the sum of FVC severity score plus the 6MWT severity score) was 4.1 (range 2 to 6), indicating the population included in this study had the attenuated form of MPS II. A subset of 53 patients were aged 12 years and above at the time of enrolment and completed the self-reported version of the HRQL measures. This subset of patients was diagnosed on average for almost 14 years and, due to the progressive nature of the disease, were a bit more severe performing worse on the clinical measures collected. Demographic and clinical characteristics of all 96 patients and the subset of 53 patients can be found in Table 1.

**Table 1:** Patient characteristics

	**Mean**	**SD**	**Median**	**Min**	**Max**	**% Missing**
**All patients (N = 96)**						
Age (yrs)	14.2	6.7	13.6	5.0	30.9	0.0
Age at diagnosis (yrs)	4.3	3.7	3.0	0.0	20.0	2.1
Duration of MPS II (yrs)	9.8	6.8	8.5	0.3	26.0	2.1
Height (cm)	127.8	14.8	126.5	99.06	170.0	12.5
Weight (kg)	35.9	13.2	32.7	19.0	78.0	7.3
6MWT (m)	400.3	100.3	407.5	37.0	588.0	2.1
% predicted FVC	56.2	14.9	54.8	16.0	90.8	2.1
Global JROM score (%)	67.3	9.1	67.8	43.4	87.2	1.0
**Patients above 12 years old who completed self-report questionnaires (N = 53)**						
Age (yrs)	19.2	4.8	18.7	12.1	30.9	0.0
Age at diagnosis (yrs)	5.5	4.5	4.0	0.0	20.0	3.8
Duration of MPS II (yrs)	13.6	6.9	12.6	0.5	26.0	3.8
Height (cm)	135.1	13.8	132.5	109.2	170.0	11.3
Weight (kg)	43.6	12.9	38.8	26.8	78.0	9.4
6MWT (m)	377.9	116.5	395.0	37.0	588.0	1.9
% predicted FVC	50.1	13.1	50.0	16.0	79.2	1.9
Global JROM score (%)	62.4	7.4	62.8	43.4	82.3	1.9

The average age of the 96 parents/caregivers who completed the parent version of the HRQL questionnaires was 42 years old and above 85% were females.

The scores of the four HRQL study measures were examined. For the subgroup of 53 patients above 12 years old, dysfunction according to the HS-FOCUS self-reported responses was found most pronounced in the Walking/Standing and Grip/Reach scores, and the Overall Function score was 0.89. Disability showed a similar pattern in the CHAQ, with scores for activities of daily living including Hygiene, Reach and Dressing and Grooming being the most affected. The average CHAQ Pain scale score was 28.22 and the average overall CHAQ score was 1.49. Results from the generic CHQ showed that distress and dysfunction were most pronounced with regards to Global Health, Physical Functioning and Role/Social-limitations–Physical, as well as Bodily Pain. Of note, very low scores were reported in the Self Esteem and Family Cohesion domains (patient self-reported scores 29 and 31, respectively), suggesting a pronounced, detrimental impact on the patients as well as their families and caregivers. Similarly, the disutility values derived in the HUI3 indicated a moderate impact, with a mean overall utility score of 0.51.

For this subgroup of patients above 12 years old, parent and patient self-reported scores were compared and parent’s ratings suggested a slightly higher level of dysfunction and distress than those of patients (data not shown). The correlation coefficient between patient self-reported and parent overall scores was found to be positive ranging from 0.69 in HUI3 to 0.81 in CHAQ.

The functional areas with higher level of dysfunction reported by parents on their 96 children were consistent with those reported by the patients themselves. The HS-FOCUS Overall Function score reported by parents was 0.74, overall CHAQ score was 0.67 and the utility score was 0.29. Similarly, the CHQ responses completed by the parents were very low, also indicating pronounced distress associated with MPS II across most of the CHQ domains.

In order to assess the validity of these questionnaires in describing the effect of MPS II on patients’ HRQL, scores were correlated with objective clinical measures. For the subgroup of patients 12 and above who completed the patient self-reported versions, the correlation coefficients between the disease-specific HS-FOCUS domains and selected clinical parameters collected in the clinical trial were found to follow the expected direction and were statistically significant at 95% level of confidence, where the strongest correlations were seen with the 6MWT, and the global JROM score (Table 2). The Overall Function scores of the HS-FOCUS were lower (indicating less degree of incapacity) for higher distance walked in 6 minutes as recorded in the 6MWT (r = −0.6) and better joint mobility as indicated by a global JROM closer to 100% (r = −0.3). The correlation between the other HRQL measures and study clinical measures followed the same expected direction indicating that patients who performed worse in the clinical parameters were consistent in reporting less quality of life as measured by the study questionnaires. The largest coefficients were found between the 6MWT results and the Activities score of the CHAQ (r = −0.6), and the JROM and the Physical Functioning of the CHQ (r = 0.5). HRQL measures were also correlated with the duration of illness since diagnosis, and pair-wise correlations using the patient self-reported responses were found to be positive and statistically significant at 95% level of confidence for the HS-FOCUS overall score, CHAQ VAS scales for pain and well-being and CHQ Global Health Score. The Overall utility score from the HUI3 was also statistically significant and negative since lower utility indicates a worse health state (data not shown).

**Table 2:** Spearman’s correlation between the patient self-reported HS-FOCUS and clinical measures

**HS-FOCUS**	**N**	**6MWT**	**% Predicted FVC**	**JROM**
Walking/Standing Function Score	48	−0.5840*	−0.1334	−0.2084
Grip/Reach Function Score	48	−0.4014*	−0.1210	−0.3376*
Sleeping Function Score	48	−0.1609	−0.1679	−0.1345
Schooling Work Function Score	44	−0.3665*	0.1008	−0.1634
Activities Function Score	48	−0.5754*	−0.1487	−0.1843
Breathing Function Score	45	−0.2219	−0.1158	−0.1842
**Overall function score**	**48**	−0.5871*	**−0.1717**	−0.2961*
Child Satisfaction Score	47	0.2511	0.1124	0.0332
Child Botheredness Score	47	−0.4207*	−0.0461	−0.2003

* Statistically significant at the 95% level of confidence.

To better understand and interpret these results, a targeted literature search was conducted in order to identify published studies that had used the same questionnaires as this study to assess aspects of HRQL in children and adolescents to be able to compare them. A total of five such publications involving the CHAQ (one article), the CHQ (four articles) and the HU13 (two articles) were identified, with some of these articles covering more than one questionnaire of interest.

The CHAQ-DIS scores reported by patients with MPS II as collected in the trial indicated a 7-fold more pronounced disability compared with healthy children, and scores were similar in severity to that experienced by patients with juvenile idiopathic arthritis [19] (CHAQ DIS scores 1.5 vs. 1.7 for MPS II) (Table 3).

**Table 3:** Patient-reported CHAQ scores compared with scores in a juvenile idiopathic arthritis population

**CHAQ**	**Juvenile idiopathic arthritis**	**MPS II**	**Healthy children**
CHAQ DIS	1.73	1.49	0.20
VAS Pain	55.60	28.20	0.00
VAS Well-being	51.60	34.40	0.00

Of the four previous published studies identified reporting CHQ scores, one included the patient-reported CHQ-CF87 scores [20], and the other three the parent-reported version CHQ-PF50 [21-23]. Normative values were also identified, based on the parent-reported version of the CHQ.

**Figure 1:**
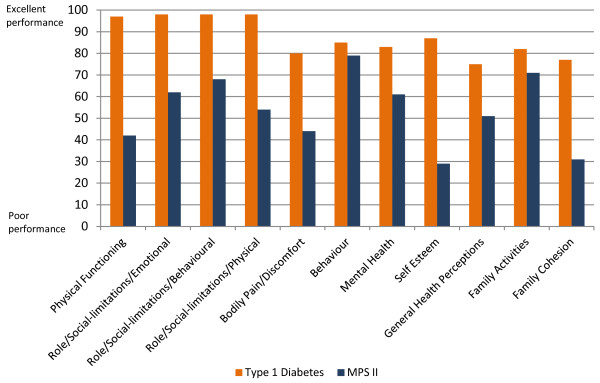
Patient-reported CHQ-CF87 scores compared with scores in a diabetic population.

**Figure 2:**
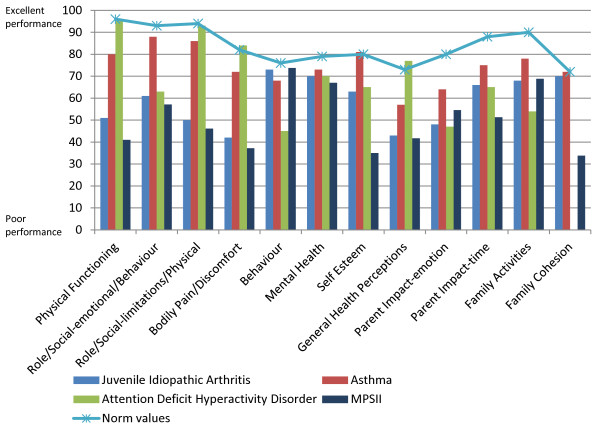
Caregiver-reported CHQ-PF50 scores compared with proxy-reported scores in other paediatric populations and with proxy-reported normative values.

In general, scores reported by patients with MPS II and their parents/caregivers were consistently lower than scores in the other paediatric populations identified (except the Behaviour score as reported by the parents); and they were also considerably lower than the normative values. Consistent with results obtained from the functional measures (as described above), the Physical Functioning and Role/Social-limitations–Physical in MPS II were low (41 and 46 as reported by parents), compared with the normative values that have been described as 96.1 and 93.6, respectively [24]. By comparison, several paediatric asthma studies have shown values in these domains of 85.5–93.7 and 86.5–92 [16]. The Self Esteem and Family Cohesion scores were lower in MPS II than in the other paediatric populations identified. Similarly, in relation to Mental Health, there was a score of 66.9 in MPS II, 69.9 in juvenile arthritis, and 69.7 in ADHD, compared to a normal reference value of 78.5 (Figures 1 and 2).

**Figure 3:**
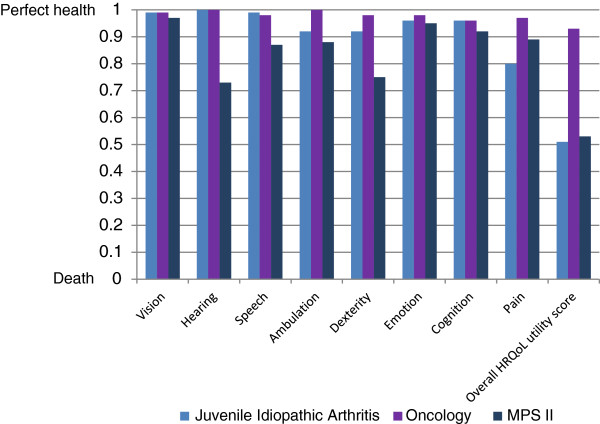
Caregiver-reported HUI3 utility values compared with caregiver-reported scores in other paediatric populations.

Comparison between the HUI3 utility scores that parents provided for their children with MPS II, with the scores for other populations, corroborated published evidence that hearing and related speech problems are a major impairment in MPS II patients [25]. Other prominent areas of impairment included dexterity and ambulation (Figure 3). MPS II scores were lower in all HUI3 attributes than scores for patients with cancer, and lower in all but the Pain scores compared to those in patients with juvenile arthritis.

## Discussion

This study shows that MPS II has a considerable impact on both the physical functioning and psychological aspects of patients’ HRQL. This dysfunction was evident in scores for different disease-specific and general instruments self-reported by patients or their parents/caregivers and these scores helped in identifying the most affected areas. Also, even though the population described (participants in clinical trial) had the attenuated form of MPS II as dictated by the trial’s inclusion criteria, their level of impairment across most dimensions (particularly Pain and Self Esteem) was greater than that of other comparable paediatric populations.

Key findings of the study included confirmation that physical function and the ability to perform day-to-day activities were the most affected areas as described by all of the study measures, with specific dysfunction with regards to dexterity and upper body function. Pain and discomfort were also amongst the most prominently affected areas of HRQL and were of comparable severity to that seen in juvenile refractory arthritis, a disease well-known for its painful and incapacitating effects. The study also corroborated published evidence on hearing impairment associated with MPS II, by showing that Hearing represented the most severe disutility among the HUI3 domains. This disability merits special attention given its potentially harmful effect on children, especially with regards to speech development and school performance if the deficit is not promptly recognised [23,25].

The results also showed a psychological impact on patients, with Self Esteem and Family Cohesion being the most affected domains, having scores even lower than those for physical function. This indicates that the disease severely affects self-image, which is particularly concerning given that a lack of self esteem is likely to have a damaging influence from childhood through adolescence and into the longer term [26]. The low scores for Family Cohesion provide an indirect indication of an important additional dimension that needs consideration when assessing the overall effect of MPS II on HRQL – the impact on parents who act as caregivers. This adverse influence was also reflected directly in responses to the parent-specific questions in the CHQ, with lower reported scores compared to those for other paediatric populations, especially with respect to the amount of time parents had for their own needs as a consequence of their child’s condition. The fact that parents or caregivers described the distress and dysfunction associated with MPS II as slightly higher compared with corresponding ratings made by patients themselves is a pattern that has been observed in several published studies of families where children have chronic diseases [27-30]. One explanation for these findings may be that whilst patients may learn to live and cope with their disease, parents have a longer term perspective and are concerned about future disability which may be incorporated in their assessment thus generating worse scores compared with patient ratings [30,31].

We believe that the present study has several key strengths. Its use of a broad battery of validated patient and caregiver questionnaires has provided a rare opportunity to describe the burden of MPS II as experienced by both patients themselves (at baseline of their participation in a clinical trial of ERT) and their caregivers, and to compare this with physiological measures (from the trial protocol). This approach has facilitated the understanding of the effects of MPS II, viewed both in isolation and, crucially, in comparison with other chronic diseases. The combined use of disease-specific and generic instruments is also a strength, and all of them strongly correlated with objective clinical indicators supporting the consistency of the results.

The HS-FOCUS questionnaire, for example, has provided detailed information on specific MPS II-associated distress and dysfunction. By contrast, the CHAQ and the CHQ are generic instruments that have been employed in numerous paediatric populations and lend themselves to comparisons of the HRQL burden across a range of chronic conditions: they have, therefore, allowed the impact on patients with MPS II to be gauged against that in peers with other disabling diseases. In addition, it is important to note the high correlation in this study between study Overall Function scores in the HS-FOCUS and objective measurements of functional capacity such as the 6MWT, as well as a moderately high correlation with the global JROM score as evidence of the validity of using these instruments for measuring the adverse influence of MPS II on patients’ lives and daily physical activities. The correlation with JROM scores represents an important finding since this clearly suggest that joint stiffness and flexion contractures can result in severe loss of Overall Function as well as Grip/Reach function in MPS II patients. Even though the correlation with lung function did not reach statistical significance, the current results show a relationship which is stronger than generally observed in e.g., chronic obstructive pulmonary disease [32,33].

Some limitations of our findings are that they are based wholly on patients with the attenuated form of MPS II, and cannot be assumed to hold for those with the severe form of the disease. However, given the nature and range of clinical problems in severe MPS II, it is reasonable to speculate that it too is associated with considerable adverse effects on the HRQL of patients and caregivers. Intelligence quotient of patients and whether patients were living in a rural or urban environment might have influenced their quality of life, however, this information was not available. Finally, the authors recognize the possible language and cultural differences between patients in the four countries where patients were recruited; however, individual country samples were too small to allow for subgroup analyses in this study.

Our study has key implications for clinical practice. Previously, there has been surprisingly little published on the impact of MPS II on patients’ HRQL, and nothing on how the disease affects parents and/or caregivers. Appropriate knowledge in these areas is obviously essential in the management of any chronic disease. This is particularly so for childhood conditions, where there are consequences not just for the individual patient but also for the HRQL of parents and other caregivers, and for family life. With regards to MPS II, this study has enabled the development of a clear and comprehensive picture of the true impact of the disease and its consequences. This deeper understanding should help paediatricians, other clinicians and other professionals in recognising the difficulties and needs of people with MPS II and their caregivers, and in targeting support and interventions for these people appropriately.

Our findings are also relevant to the advent of ERT as a candidate for standard treatment of patients with MPS II. This use has been advocated strongly by some opinion leaders [34]. In reality, however, published trials of the drug to date have provided only very limited evidence on humanistic outcomes such as a range of important aspects of HRQL [7-10]. Hence, it is reassuring that our study found a clear association between key clinical function measures for MPS II at baseline in one of these trials and contemporaneous scores on HRQL instruments. It follows that use of such instruments to assess changes in HRQL should be a key part of further evaluation of ERT. Justification of its routine use in a disease as rare as MPS II will require a clear understanding of how the HRQL of patients with MPS II and their caregivers may benefit from the treatment, through thorough and accurate evaluation over time. These results have implications for other MPSs where similar results may be found and conducting similar studies would be important too. Other justifications include health economic evidence on ERT, including assessments that balance the cost of illness (which may be substantial) against those of the value of the treatment. Ultimately, if ERT is confirmed as standard therapy for MPS II, it will be crucial to facilitate access to the treatment by ensuring that health care providers are made more aware of how this orphan disease affects patients and caregivers in both the short and long term. In this broad context, and for all these reasons, it is hard to overestimate the importance of collecting patient data to describe accurately the distress and dysfunction associated with MPS II – the focus of our study.

## Conclusion

In summary, this study demonstrates that it is possible to quantify the HRQL impact of a rare disease by employing standard validated questionnaires as a metric for quality of life, including preference-based measures to capture the disutility of a condition. The HRQL impact has been described in detail for a group of patients with MPS II at entry to a clinical trial and compared to physiological measures gathered in that study, and with relevant other chronic paediatric diseases. Findings showed that MPS II has a considerable impact on both the physical functioning and psychological aspects of patients’ HRQL, with a higher level of impairment across most dimensions (particularly Pain and Self Esteem) than that of other comparable paediatric populations. Such humanistic data are an increasingly important influence in the establishment of priorities for health care spending, and as a component of health economic analysis.

## Abbreviations

6MWT: Six-Minute Walk Test; BP: Bodily Pain; BE: General Behaviour; CH: Change in Health; CHAQ: Childhood Health Assessment Questionnaire; CHAQ DIS: CHAQ Disability Index Score; CHQ: Childhood Health Questionnaire; ERT: Enzyme Replacement Therapy; FA: Family Activities; FC: Family Cohesion; FVC: Forced Vital Capacity; GAGs: Glycosaminoglycans; GH: General Health perceptions; HRQL: Health-Related Quality of Life; HUI: Health Utilities Index; HS-FOCUS: Hunter Syndrome-Functional Outcomes for Clinical Understanding Scale; JROM: Joint Range Of Motion; MH: Mental Health; MPS II: Mucopolysaccharidosis type II; PE: Parental impact-Emotional; PF: Physical Functioning; PT: Parental impact-Time; RB: Role/social-Behavioural; RE: Role/social-Emotional; RP: Role/social-Physical; SE: Self Esteem; WHO-ICIDH: World Health Organization International Classification of Impairment, Disability and Handicap.

## Competing interests

**Mireia Raluy-Callado** is employed by Evidera, which provides consulting and other research services to pharmaceutical, device, government and non-government organisations. In this salaried position, Mireia Raluy-Callado works with a variety of companies and organisations. She receives no payment or honoraria directly from these organisations for services rendered.

**Wen-Hung Chen** is employed by Evidera, which provides consulting and other research services to pharmaceutical, device, government and non-government organisations. In this salaried position, Wen-Hung Chen works with a variety of companies and organisations. He receives no payment or honoraria directly from these organisations for services rendered.

**David A H Whiteman** is a full-time employee of, and stockholder in, Shire Human Genetic Therapies. He has been the medical lead on the MPS II (Hunter syndrome) therapeutic (Elaprase) development program for the past 7 years.

**Juanzhi Fang** was a full-time employee of, and stockholder in, Shire Human Genetic Therapies when this manuscript was developed.

**Ingela Wiklund** is employed by Evidera, which provides consulting and other research services to pharmaceutical, device, government and non-government organisations. In this salaried position, Ingela Wiklund works with a variety of companies and organisations. She receives no payment or honoraria directly from these organisations for services rendered.

## Authors’ contributions

DAHW and JF contributed to acquisition and interpretation of data from the clinical trial. MRC, WHC and IW contributed to the conception and design of the study and interpretation of the data. IW designed and performed the literature review; MRC and WHC carried out the statistical analyses. All authors contributed to drafting the article or reviewing and revising it critically for important intellectual content; all authors approved the final version to be published.
